# Vaginal Examinations During Childbirth: Consent, Coercion and COVID-19

**DOI:** 10.1007/s10691-021-09453-7

**Published:** 2021-04-08

**Authors:** Anna Nelson

**Affiliations:** Department of Law, Centre for Social Ethics and Policy, University of Manchester, Manchester, UK

**Keywords:** Autonomy, Choice in childbirth, Consent, COVID-19, Pregnancy, Vaginal examinations

## Abstract

In this paper I assess the labour ward admission policies introduced by some National Health Service (NHS) trusts during the COVID-19 pandemic, arguing that these intersected with other policies in a manner which may have coerced birthing people into consenting to vaginal examinations they might have otherwise refused. In order to fully understand the potential severity of these policies, I situate this critique in the historical and contemporary context of the problematic relationship between consent and vaginal examinations. Identifying the legal wrongs associated with performing coerced vaginal examinations, I highlight that the law is inadequately equipped to provide appropriate redress. Further, I illustrate that the issue explored in this paper reflects broader problems which exist with regard to the focus of, and the (under)investment in, the maternity services.

## Introduction

During the height of the COVID-19 pandemic in the UK, I wrote a blog raising concerns that the policies being implemented by some National Health Service (NHS) trusts might have the effect of coercing birthing people into consenting to vaginal examinations (VEs) with which they were not comfortable (Nelson 2020). These policies stated that only once labour had been ‘confirmed as established’ would a pregnant person^1^ be admitted to the labour ward. Viewed discretely, they may seem an innocuous response aimed at reducing risks of COVID-19 transmission in hospitals. However, the coercive effect occurred because these policies sat alongside two others: a COVID-19-specific policy that only upon admission to the labour ward would a pregnant person’s birth partner be allowed to join them, and the existing routine use of VEs to confirm labour. The traction the blog received amongst organisations, midwives and individuals who work tirelessly to ensure that the rights of birthing people are protected both surprised me, and indicated that this concern was part of a broader issue which predated COVID-19. Reflecting upon this, it is important to situate my context-specific analysis of VEs within the wider context of insufficiently consented/unconsented VEs, and of the broader problems facing the maternity service, in order to understand the true significance of the COVID-19 admission policies (CAPs, hereafter).

I begin by outlining how the CAPs may have had the effect of coercing consent to VEs, even though this was not their intended purpose, before briefly exploring the historical and contemporary context of the problematic relationship between consent and VEs. Doing so allows the assessment of the CAPs and their impact to be situated in the appropriate socio-legal context and facilitates a deeper understanding of their problematic nature. I then consider the harms associated with these VEs, and highlight the difficulties that people are likely to face when attempting to seek appropriate legal redress. In doing so, I expose some of the broader, institutional problems which underpin the ongoing issues with unconsented VEs, and explain why these facilitated the exacerbation of this issue by the CAPs put in place in response to the pandemic.

## The COVID-19 Admission Policies: Understanding the Problem

In response to COVID-19, many NHS trusts sought to limit the volume of people entering hospital wards, to help control the spread of the virus. As part of this, some implemented policies which dictated that *only* upon the confirmation of established labour would a birthing person be admitted to the labour ward. Those who presented at the hospital prior to this stage had two options: wait for established labour to begin in the antenatal ward or return home until labour progressed. Crucially however, this policy sat alongside the position adopted by these (and most other) NHS trusts that *only* once a person was admitted to the *labour* ward would their birth partner be allowed to join them. Therefore, not only was the confirmation of established labour the ‘gatekeeper’ to the labour ward; it was also necessary for a birthing person in hospital to be allowed to be joined by their birth partner.

Implementing policies which reduced COVID-19 transmission risk was understandable and necessary during the pandemic. However, such policies still ought to have been created and communicated in a manner which ensured that the rights of birthing people were protected, which unfortunately was not the case in this instance. Though the policies which made admission to the labour ward and the right to have one’s birth partner present contingent on ‘confirming established labour’ were not inherently problematic, they became so when viewed in combination with the fact that the routine, accepted protocol for confirming established labour was the use of a VE (Nelson 2020). This resulted in some birthing people believing that if they wanted admission to the labour ward, which was necessary to enable their birthing partner to join them in hospital, they *had* to consent to a VE, as this was the only way of confirming labour.

In reality, there are other methods which can be used to confirm labour,^2^ but if these were not communicated clearly, there is a risk that pregnant people believed consenting to a VE was the *only* way to ensure their birth partner was allowed to join them. Therefore, the coexistence of the CAPs and the routine protocol to confirm labour using a VE may have resulted in pregnant people feeling coerced into consenting.

Sadly, there is now evidence that these fears were well-founded, with research demonstrating that, of the women who gave birth in a hospital which prevented partners attending until they were in established labour, 17.4% said they felt forced to have a vaginal examination and 82% cited that this felt like a requirement so that they could be reunited with their partner (Birthrights 2020). Writing in *The Telegraph*, Hill (2020) adds real voices to these statistics, voices such as Ruth’s: “I tried to refuse VE but the hospital threatened to send my partner home. In the end I gave in and let them, but it felt very violating.”

To fully understand the potentially coercive nature of these CAPs, it is vital to outline the importance of the birth partner, to which birthing people had *no* access until they were admitted onto the labour ward. The importance of having a trusted partner present throughout birth has been repeatedly reaffirmed by bodies such as the World Health Organization [WHO (2020)] and the Royal College of Midwives (RCM), who noted that this “is known to make a significant difference to the safety and well-being of women in childbirth. At times like this, when coronavirus is heightening anxiety, that reassurance is more important than ever” (RCM 2020a, 1).

Even during an unprecedented situation such as COVID-19, any measures implemented by the NHS which infringe upon the rights and autonomy of birthing people must be proportionate and justifiable, and any application of the CAPs which effectively rendered a VE necessary (or perceived them as necessary) cannot meet these standards. While many perceive VEs as the simplest method for confirming established labour, there are others available. For example, “huge amounts of information can be gathered by just listening and watching”, as the pregnant person’s behaviour can provide midwives with great insight about the progress of labour (AIMS 2017). Alternatively, on some birthing people a purplish line appears during labour, developing upwards from the anus—the length of which often roughly corresponds to dilation (AIMS 2017).

Indeed, some midwives oppose the routine use of VEs, claiming that these are neither desirable nor justifiable as they are often uncomfortable for the birthing person (Dixon 2005, 23), are imprecise and ineffective (Huhn and Brost 2004; Shepard et al. 2010, 1) and increase risk of infection (Dixon and Foureur 2010, 24; Dahlen et al. 2013). The ongoing commitment to their use, some suggest, is due to a “specific, entrenched labour culture” which centres on a “medicalised perspective on childbirth” (Reed 2015; Shabot 2020, 6). The issue of medicalisation is one which appears regularly in the discourse around pregnancy and childbirth, and has been repeatedly linked to the denial of autonomy in childbirth.^3^

## The Troubled Relationship Between the Medical Vaginal Examination and Consent: The Magnifying Impact of the Pandemic

Understanding the context against which the CAPs were implemented is important, as it explains why fears about their coercive impacts were well-founded; there is a long history within the medical profession of not only tolerating, but also defending (Cardoza 1992), the practice of performing VEs without appropriate consent.

The development of the modern maternity system, where birth usually takes place in hospital with medical oversight, has resulted in ‘obstetric hegemony’ which expects birthing people to be compliant with whatever testing and surveillance is deemed necessary by medical professionals (Anderson 2004; Milne 2019, 157). Though this paternalistic, ‘doctor knows best’ attitude was a feature across medicine, it was—and to an extent remains—particularly pervasive within the maternity setting, where it is reinforced by the societal expectation that pregnant people should do whatever it takes to maximise foetal well-being (Purvis 2017). The result is an *assumption* that all birthing people will submit to the dominant procedures and practices, with those who push back against these (for example, by refusing consent to a VE) being labelled “difficult” or “irresponsible” (Douglas 1991, 173; Meredith 2005, 112). This also ties into the wider (gendered) issue that women who assert opinions contrary to those in positions of power are often portrayed as unreasonable or hysterical. As one midwife commented: “women are made to feel so terrible if they don’t conform…if you don’t conform you are in a way stereotyped into being a bad woman” (Birthrights 2013, 16). This normalised situations in which consent to procedures during childbirth was simply assumed, or where birthing people were subjected to extensive pressure and coercion from staff to provide consent.

Consequently, until fairly recently, significant numbers of birthing people had VEs imposed upon them during labour absent valid consent. Some were simply never asked for consent to the VE, an issue borne out of pervasive systematic assumptions that all birthing people ought to adhere to standard medical procedures. Similarly, some only consented as a result of experiencing considerable pressure from staff attending their birth (Hill 2019). Again, we see the impact of a societal narrative which assumes that the ‘good mother’^4^ must subvert their own will or concerns in order to do what (medical professionals deem) is best for the baby.^5^ Most worryingly, in a number of instances, explicit refusal of consent was ignored (Hill 2015). For example, one woman reported, “I knew I could refuse. I put it in my birth plan. But that was never read…they just did them without my consent. Consent in childbirth is a joke” (Hill 2015, np).

This highlights egregious failures to understand the implications of performing such an intimate and invasive procedure absent valid consent, and of making people feel like they are not in control of their birth and their bodies. However, there has been a concerted effort over the past decade or so to ensure that dignity and respect are afforded during childbirth—which has resulted in a dramatic reduction in VEs being performed without adequate consent (Birthrights 2013).

Despite these significant improvements, issues of obstetric compulsion and insufficient consent in relation to VEs still persisted pre-pandemic. Requests for consent are not always communicated sufficiently clearly; when language is too vague and euphemistic, birthing people may not actually understand what they are consenting to, with the result that they feel like they have been subjected to an unconsented VE (Birthrights 2013, 16). There are also concerns that people are still *expected* to consent to certain procedures during birth and face coercion from staff if they attempt to refuse (Hill 2019). Further, as will be demonstrated in the next section, healthcare professionals are fairly immune from any legal liability where unconsented/inadequately consented examinations do occur (Pickles and Herring 2020).

Unconsented VEs also occur in other areas of medical practice. For a long time it was commonplace for medical students to ‘practise’ VEs on patients anaesthetised for routine gynaecological operations, who had never been asked for their consent (Stepney 1994). Though there is now a greater understanding of the problematic nature of such conduct, research indicates that some medical students continue to perform intimate examinations on anaesthetised patients without any consent (Friesen 2018), and that “on many occasions, more than one student [had] examined the same patient” (Coldicott et al. 2003, 97). This illustrates a continuing failure within parts of the medical profession to understand the significance and consequences of performing VEs absent consent.

That it is known that unconsented VEs continue to be performed and yet those involved routinely avoid any form of liability exposes wider, institutional problems in relation to both accountability,^6^ and the way that women—particularly birthing women—are viewed and devalued within the medical system.^7^ It clearly demonstrates that the frameworks which allowed unconsented VEs to occur so regularly have yet to be fully dismantled (Pickles 2020, 141), and in doing so it underscores the credibility of the threat posed by the CAPs to the autonomy of birthing people.

When we view the CAPs in light of both the fact that unconsented VEs were fairly routine until quite recently and the fact that the frameworks which enable these to occur have yet to be fully dismantled, we can understand why they invoked such concern. Past experience provided people with good reason to believe that the potentially coercive nature of these CAPs would be borne out in reality. It also means that many people may have a deep personal understanding of the harm which can be associated with VEs that occur absent valid consent.

Further, by exacerbating and formalising the existent problem of coercive pressure to consent to VEs in labour, the CAPs shone a spotlight on the issue, providing those who had ongoing concerns about obstetric compulsion with a clear path to bring these concerns to a receptive audience. I suspect that the traction my blog received reflects this: by couching the issue of unconsented VEs in the COVID-19 context, I made a pre-existing issue ‘current’ and thus provided a highly visible vehicle for people to express their long-held concerns.

COVID-19 has not created the structures in which coerced VEs are able to occur; rather, it has exacerbated a problem that already existed. By understanding the context against which the CAPs were introduced, we can more fully comprehend the gravitas of the concern that these would result in an actual increase in VEs being performed absent valid consent.

## Facilitating harm, impeding redress

### Harm

Ensuring people feel in control of their birth is vitally important, as “denying [birthing people] choices about childbirth forces them to relinquish their identity by surrendering to certain life-altering experiences over others” (Romanis 2019, 255). When a person has to endure a VE to which they did not validly consent, they are not only denied control over their own birth, but they are also subject to an attack on their dignity. Resultantly, policies which fail to centre and protect the well-being of the birthing person can have long-term, negative implications for them. Indeed, “feelings of loss of control” and a “lack of dignity” (The Birth Trauma Association 2018, np) in birth, feelings which will both be evoked by the performance of an unconsented/coerced VE, are strongly associated with post-birth trauma and even post-traumatic stress disorder (PTSD) (The Birth Trauma Association 2018). This is particularly true given both the intimate nature of VEs and the gravitas of the reasons why some may wish to reject these—such as experience of sexual assault or previous medical or birth-related trauma (AIMS 2017).

While this is, of course, true of unconsented VEs whenever they occur, there are two reasons why the impact has been heightened by the pandemic. The first is that the negative mental health impact of experiencing an unconsented VE will likely have compounded at this time by the wider implications of the pandemic. For a range of reasons—uncertainty, changing birth plans,^8^ rapidly changing hospital policies, fears about the risk COVID-19 posed to their foetuses/newborns—many people who were pregnant or who gave birth during this period felt increased anxiety about this^9^ (Birthrights 2020). Further, the lockdown may have resulted in people finding negative/traumatic experiences harder to cope with and recover from because social support networks were extremely limited.^10^ Not only does this compound the immediate impact of obstetric trauma; it may also prolong recovery.

The second issue is a legal one. The CAPs went beyond simply facilitating the ongoing issue of inadequately consented/unconsented VEs; they actively increased the likelihood of these being performed. This amounts to not simply a failure to protect the autonomy and dignity of the birthing people, but also a breach of the NHS’ human rights obligations. Under the Human Rights Act,^11^ the NHS is bound to act in a manner compatible with the European Convention on Human Rights (ECHR). Article 8 of the Convention provides citizens with the right to private and family life, and covers matters pertaining to “the physical and psychological integrity of a person”,^12^ including the requirement that “free and informed consent” is obtained prior to medical treatment or examination (Council of Europe 2015). Though interferences with Article 8 can be justified where these are necessary ‘for the *protection of health*’,^13^ it cannot be said that policies which significantly increase the likelihood of unconsented VEs being performed protect health, where health is (correctly) viewed holistically as encompassing both the mental and the physical.

Additionally, the CAPs may have effectively precluded some people from accessing the maternity care they desired. If someone was unwilling to consent to a VE but wanted to ensure their birth partner was able to be present during labour, they may have felt they had no option but to birth at home (Romanis and Anna 2020, 7). As many trusts suspended their homebirth services, this may have meant they saw no option but to birth without any medical attendance (Summers 2020a, b).

The CAPs have resulted in VEs gaining a ‘gatekeeping’ role, and this has compounded the coercive pressure to consent to these, thereby increasing the frequency with which VEs are performed absent valid consent. Therefore, we have a situation in which policies have been introduced which not only fail to prevent a harmful practice from occurring, but which actually increase the likelihood of this practice. This is not only inherently problematic; it is also incompatible with the NHS’ human rights obligations under the ECHR.

It is important to reflect on why these CAPs were implemented given their potential to perpetuate harmful practices. Of course, the need to rapidly implement measures to deal with an unprecedented situation will have limited the capacity for review and evaluation of proposed policies. However, it seems likely that this oversight is rooted in something deeper than simply the need for quick action to curb the pandemic. It raises an important concern about the lens through which maternity policies are scrutinised, with efficiency appearing to be the key aim, rather than the protection of the rights and well-being of the birthing person. This reflects both the ongoing systemic and cultural tendency to see the dignity and autonomy of the birthing person as of secondary importance to the birth of a healthy baby, and the chronic underinvestment in the maternity services.

This latter issue predates the pandemic, and has created a self-perpetuating cycle of poor pay, staff shortages and low morale/job satisfaction (RCM 2020b; c). Where units are understaffed (as they are across the board), the need for efficiency is elevated, and anything which is not seen as central to the core aims of the service is likely to be neglected as a result. Where this underinvestment is coupled with the ongoing systemic failure to recognise the importance of rights-centred maternity care, it is easy to see how the importance of ensuring the dignity and autonomy of the individual birthing person gets superseded by other considerations. The impact of (long-term) underinvestment has also been seen elsewhere in the pandemic response; when homebirthing services were suspended and midwife-led units closed by some trusts, short-staffing was given as a key reason (Sherwood 2020; Davis 2020). Here again, we see an illustration of the fact that though the pandemic may have amplified problems within the maternity service, it did not create them. Rather, it has served to “expose the gaps that already exist” (RCM 2020d).

It is also vital to acknowledge that the negative impact of these CAPs will not have been felt equally by all. Although disaggregated data regarding VEs in the COVID-19 context is not yet available, we know that both race and socio-economic circumstances can have a significant impact on the maternity care a person receives (MBRACE-UK 2019). Black women are five times more likely to die during pregnancy, birth or the post-partum period, and Asian women twice as likely (MBRACE-UK 2019). Though the exact causes of this are yet to be fully understood (McKenzie 2019), it is clear that these statistics are underpinned by racism ingrained within the hierarchical medical framework (Ekechi, in Randone 2020).

There is a great deal of testimonial evidence indicating that black people are less likely to be listened to or taken seriously when they raise concerns during pregnancy and birth (FIVEXMORE 2020). As a result, structural biases and unconscious staff biases have a chilling effect on the ability of these people to advocate effectively for themselves during pregnancy. A further consequence is that it creates a situation in which some black people refrain people from voicing their concerns at all (Kasprzak 2019), out of fear that this will have no impact other than to result in them being inappropriately labelled as “difficult” or stereotyped into the racist trope of the “angry black woman” (Ashley 2014, 28).

There is also a disparity in access to information about one’s rights and choices during childbirth; both black people (FIVEXMORE 2019) and those from lower socio-economic groups are “prone to have lower awareness of… their rights and choices about the maternity care to which they were entitled” (Birthrights 2013, 17). This means that these groups are less likely to be aware that they have the right to refuse to consent to VE and are also more likely to be unaware that failure to respect their refusal is a legal wrong. Where they do attempt to seek redress, the general hurdles associated with holding healthcare professionals to account for performing unconsented VEs will be significantly heightened by the racism and unconscious bias ingrained within the healthcare systems.

Therefore, it is reasonable to hypothesise that the CAPs are likely to have disproportionately impacted those who are from marginalised groups, and that these people may struggle the most to get their voices heard when raising concerns about the treatment they experienced.

### Redress

Theoretically, those who were subject to VEs on the basis of defective consent due to the coercive impact of the CAPs could seek legal redress for this; something which individuals may find valuable in terms of seeking accountability, and for validating the extent of the harm done to them. In recognition of the importance of autonomy, the law dictates that it is “a criminal and tortious assault to perform physically invasive medical treatment…without the patient’s consent”.^14^ Further, it is clear that this consent must be informed and freely given—as coerced consent is not valid consent (Wolf and Charles 2018).

However, the reality is that those who do seek to bring legal action—whether through tort or criminal law—will face a number of barriers. While, in theory, a claim could be brought in tort for either negligence or battery, there are a number of significant hurdles which would be faced by a claimant trying to bring an action under either of these heads. The success of both actions rests on the fact that consent was coerced, rather than freely given. Proving that, on the balance of probabilities, their consent was coerced to the extent which rendered it invalid, could prove challenging for the claimant. A further obstacle faced in both instances is that tortious ‘harm’ is measured in relation to monetary compensation. This could be problematic as the harm that occurs when an invalidly consented VE is performed is of a kind which would be very difficult to quantify in a manner which would allow for redress under tort law. The attempt to establish negligence would face a further challenge when it comes to satisfying the casual element of the tort. Proving causation in medical negligence cases is “notoriously” complex,^15^ and it is likely that a claimant may struggle to satisfy the court that, *but for* the coercion, they would not have consented to a VE.

A medical professional can also be charged with battery under the criminal law where they “intentionally or recklessly touch the patient without consent” (Herring 2002, 153). However, despite the fact that “there seems to be little reason to question the applicability of the crime of battery to unauthorised vaginal examinations during labour”, this clear applicability “does not translate into this crime being applied within the maternity care context” (Pickles 2020, 138). This might, in part, be due to judicial reticence to reprimand doctors under the criminal law, save in circumstances where “the professional was acting maliciously” (Herring 2002, 153). However, it is important to recognise that in the case of unconsented VEs, real and significant harm can be caused regardless of the intentions of the medical professional and that medical professionals ought to be very aware that “consent is a fundamental legal and ethical principle” which must be respected (General Medical Council 2020, 4).

The fact that not only does the practice of performing VEs absent appropriate consent continue, but also that policies which actually increase the likelihood of these occurring are able to be implemented, demonstrates the ineffectiveness of the law as a tool to tackle this issue (Pickles 2020, 138). This is disempowering and invalidating for those who experience harm as a result of these policies and practices, and it also reflects on a broader problem of accountability that pervades the medical institution (Erdman 2015, 48). In the maternity setting, this issue is further compounded both by the fact that there continues to be gendered devaluing of women in society (Pickles 2020, 141) and the tendency to see the birth of a ‘healthy baby’ as the central focus of maternity care, regardless of the way the birthing person experiences that birth.

## Conclusion

COVID-19 hugely impacted all aspects of medical care, and the maternity services were no exception. The rapid introduction of policies and practices aimed at protecting staff and birthing people was understandable and laudable. However, the CAPs addressed in this article, in combination with the routine use of VEs to establish confirmed labour, did not strike an appropriate balance between public health concerns and protecting the rights and autonomy of birthing people. They created a real risk that people would be coerced into consenting to VEs which they were not comfortable with, as they felt this was the only way to access both the labour ward and their birth partner.

The implementation of the CAPs, and the failure to appreciate the potential consequences of these, also acts as a mirror to reflect some of the wider problems facing the maternity service, both during the pandemic and beyond. The issues pertaining to VEs reflect an institutional failure to understand the significance and importance of quality, rights-centric maternity care, which in turn is both a symptom and a cause of the chronic underinvestment in the maternity services. Where maternity units are understaffed and under-resourced, the central focus becomes efficiency. When coupled with the societal and institutional tendency to value the outcome of a ‘healthy baby’ above all else, rather than recognising that *both* the health of the baby and the protection of the holistic well-being of the birthing person are vital, this can result in policies and practices which undermine the autonomy and dignity of the birthing person. Having such policies in place both harms birthing people and serves to compound the problems facing the maternity service. The vast majority of those delivering maternity care recognise the importance of ensuring a good outcome for both the baby and the birthing person, and having to work within a system which limits their ability to ensure that the care they offer achieves this undoubtedly contributes to low morale, which in turn may be a factor in people’s decision to leave the profession (RCM 2020c).

The pandemic has solidified the perception of maternity as a ‘Cinderella service’ which is underappreciated and underfunded. However, at the same time it has thrown the problems caused by this underinvestment into stark relief—and people have taken notice. Hopefully, when the pandemic subsides, it will leave in its wake a higher, more visible platform from which to campaign for proper investment in the maternity services.

## Footnotes

1 Throughout this paper I use the terminology ‘pregnant person’ and ‘birthing person’, rather than using the gendered terms ‘pregnant women’ and ‘birthing women’. This is in recognition of the fact that not all who birth identify as a woman, and not all women have the physiology to birth. I believe this use of inclusive language is important, as those who exist outside the gender binary are most likely to be marginalised in healthcare, and thus literature aimed at protecting the rights of those accessing healthcare needs to explicitly include them. Where gendered language is used in this paper (for example, to reflect the language a specific organisation uses or to acknowledge structural/historical gendered discrimination), this will be highlighted and justified.

2 I return to this point later in the article.

3 There is a significant body of literature exploring the negative impact of medicalisation/medical hegemony over labour on the autonomy of the birthing person. See, for example, Johanson et al. (2002); Burrow (2012); Wolf and Charles (2018); Romanis et al. (2020). I also explore these themes in co-authored work that is under review elsewhere.

4 I use gendered language here as this situation has specific links to gendered oppression, connected to societal ideals and expectations about the behaviour of women and of mothers.

5 For example, Schiller talks about the coercive force of the phrase “a healthy baby is all that matters”; see, Murphy (2016).

6 For more on this issue see Pickles (2020).

7 The word ‘woman’ is used here in recognition of the fact that this is a gendered issue. There is growing recognition that there is gender disparity in medical care. See, for example, Schopen (2017).

8 For example, many NHS trusts stopped offering homebirths and water births during the pandemic, and there were fears that some trusts were using the pandemic as reason to deny some people access to maternal-request caesarean sections.

9 I make this point in co-authored work which is currently under review elsewhere.

10 For example, for a number of months the restrictions enacted in response to the pandemic meant that visits from family and friends who did not live in the same household as the birthing person were prohibited.

11 Human Rights Act 1998, s6.

12 *Y.F. v Turkey* [2003] ECHR 391.

13 Emphasis added.

14 *Re MB* [1997] 2 FLR 426 (CA) as per Butler-Sloss, LJ at [17].

15 *Fairchild v Glenhaven Funeral Services Ltd [*2002] UKHL 22, as per Lord Scarman at [69].

## References

[CR1] AIMS. 2017. Vaginal Examinations in Labour. https://www.aims.org.uk/information/item/vaginal-examinations-in-labour. Accessed 20 March 2020.

[CR2] Anderson Tricia, Kirkham Mavis (2004). The misleading myth of choice: The continuing oppression of women in childbirth. Informed choice in maternity care.

[CR3] Ashley Wendy (2014). The Angry Black Woman: The Impact of Pejorative Stereotypes on Psychotherapy with Black Women. Social Work in Public Health.

[CR4] Birthrights. 2013. Dignity in Childbirth: The dignity survey 2014: Women’s and Midwives’ Experiences of UK Maternity Care. https://birthrights.org.uk/wp-content/uploads/2013/10/Birthrights-Dignity-Survey-1.pdf. Accessed 1 December 2020.

[CR5] Birthrights. 2020. Long term impact of visiting restrictions “could be catastrophic” MPs, academics and campaigners warn NHS England CEO. https://www.birthrights.org.uk/2020/11/15/long-term-impact-of-visiting-restrictions-could-be-catastrophic-mps-academics-and-campaigners-warn-nhs-england-ceo/. Accessed 2 December 2020.

[CR6] Burrow, Sylvia. 2012. Reproductive autonomy and reproductive technology. *Teche: Research in Philosophy and Technology* 16: 31–44.

[CR7] Cardoza Linda (1992). Letters: Teaching vaginal examination. British Medical Journal.

[CR8] Coldicott Yvette (2003). The ethics of intimate examinations–teaching tomorrow’s doctors (Education and debate). British Medical Journal.

[CR9] Council of Europe. 2015. Thematic report: Health-related issues in the case-law of the European Court of Human Rights. https://www.echr.coe.int/Documents/Research_report_health.pdf. Accessed 1 July 2020.

[CR10] Dahlen Hannah (2013). Vaginal examination during normal labor: Routine examination or routine intervention?. International Journal of Childbirth.

[CR11] Davis, Nicola. 2020. NHS trusts begin suspending home births due to coronavirus. *The Guardian,* 27 March. https://www.theguardian.com/world/2020/mar/27/nhs-trusts-suspending-home-births-coronavirus. Accessed 25 May 2020.

[CR12] Dixon Lesley (2005). Building a picture of labour: How midwives use vaginal examinations during labour. New Zealand College of Midwives Journal.

[CR13] Dixon Lesley, Foureur Maralyn (2010). The vaginal examination during labour: Is it of benefit or harm?. New Zealand College of Midwives Journal.

[CR14] Douglas Gillian (1991). Law, Fertility and Reproduction.

[CR15] Erdman Joanna (2015). Bioethics, human rights, and childbirth. Health and Human Rights Journal.

[CR16] FIVEXMORE. 2019. Briefing pack. https://www.fivexmore.com/briefing-pack. Accessed 1 December 2020.

[CR17] FIVEXMORE. 2020. FIVEXMORE blog. https://www.fivexmore.com/blog. Accessed 1 December 2020.

[CR18] Friesen Pheobe (2018). Educational pelvic exams on anesthetized women: Why consent matters’. Bioethics.

[CR19] General Medical Council. 2020. Decision Making and Consent. https://www.gmc-uk.org/-/media/documents/gmc-guidance-for-doctors---decision-making-and-consent-english_pdf-84191055.pdf?la=en&hash=BE327A1C584627D12BC51F66E790443F0E0651DA. Accessed 4 December 2020.

[CR20] Herring Jonathon (2002). Medical Law and Ethics.

[CR21] Hill, Milli. 2015. ‘Consent in childbirth is a joke’: How British women are silenced in the delivery room. *The Telegraph,* 1 May. https://www.telegraph.co.uk/women/womens-health/11574412/British-women-Consent-during-childbirth-is-a-joke.html. Accessed 20 March 2020.

[CR22] Hill, Milli. 2019. Why we need to talk about #MeToo in the birth room. *The Independent,* 30 August*.* https://www.independent.co.uk/life-style/women/metoo-birth-room-consent-give-birth-feminist-milli-hill-a9069281.html. Accessed 20 March 2020.

[CR23] Hill, Milli. 2020. How pregnant women are feeling forced into ‘violating’ vaginal examinations during COVID. *The Telegraph*, 20 November. https://www.telegraph.co.uk/women/life/pregnant-women-feeling-forced-violating-vaginal-examinations/. Accessed 1 December 2020.

[CR24] Huhn Kathleen, Brost Brian (2004). Accuracy of simulated cervical dilatation and effacement measurements among practitioners. American Journal of Obstetrics and Gynecology.

[CR25] Johanson Richard (2002). Has the medicalisation of pregnancy gone too far?. British Medical Journal.

[CR26] Kasprzak, Emma. 2019. Why are black mothers at more risk of dying? *BBC News,* 12 April. https://www.bbc.co.uk/news/uk-england-47115305. Accessed 5 December 2020.

[CR27] MBRACE-UK. 2019. Saving Lives, Improving Mothers’ Care Lessons learned to inform maternity care from the UK and Ireland Confidential Enquiries into Maternal Deaths and Morbidity 2015–17. https://www.npeu.ox.ac.uk/assets/downloads/mbrrace-uk/reports/MBRRACE-UK%20Maternal%20Report%202019%20-%20WEB%20VERSION.pdf. Accessed 3 December 2020.

[CR28] McKenzie, Gemma. 2019. MBRACE and the disproportionate number of BAME deaths. *Aims Journal* 31(2).

[CR29] Meredith Sheena (2005). Policing Pregnant: The Law and Ethics of Obstetric Conflict.

[CR30] Milne Emma (2019). Concealment of birth: time to repeal a 200-year-old “convenient stop-gap”?. Feminist Legal Studies.

[CR31] Murphy Madeleine (2016). Maternal autonomy: Conference report. British Journal of Midwifery.

[CR32] Nelson, Anna. 2020. Vaginal Examinations, Consent and COVID-19. *BMJ Sexual and Reproductive Health*, 22 May. https://blogs.bmj.com/bmjsrh/2020/05/22/ve-consent-covid/. Accessed 22 May 2020.

[CR33] Pickles Camilla, Pickles Camilla, Herring Jonathan (2020). When 'battery' is not enough: exposing the gaps in unauthorised vaginal examinations during labour as a crime of battery. Women’s Birthing Bodies and the Law Unauthorised Intimate Examinations, Power and Vulnerability.

[CR34] Purvis Dara (2017). The rules of maternity. Tennessee Law Review.

[CR35] Randone, Amanda. 2020. Black mothers are five times more likely to die during childbirth. That needs to change. *Vogue,* 25 July. https://www.vogue.co.uk/beauty/article/black-maternal-mortality. Accessed 29 November 2020.

[CR36] Reed, Rachel. 2015. Vaginal examinations: A symptom of a cervical-centric birth culture. *Midwife Thinking,* 2 May. https://midwifethinking.com/2015/05/02/vaginal-examinations-a-symptom-of-a-cervix-centric-birth-culture/. Accessed 15 May 2020.

[CR37] Romanis Elizabeth C (2019). Why the elective caesarean lottery is ethically impermissible. Health Care Analysis.

[CR38] Romanis, Elizabeth C. et al. 2020. Re-viewing the womb. *Journal of Medical Ethics:* online first.

[CR39] Romanis, Elizabeth & Nelson Anna. 2020. Homebirthing in the United Kingdom during COVID-19. *Medical Law International:* online first.

[CR40] Royal College of Midwives (RCM). 2020a. Birth partners. https://www.rcm.org.uk/media/3887/birth-partners.pdf. Accessed 20 April 2020.

[CR41] Royal College of Midwives (RCM). 2020b. ‘Deliver a decent deal’ say midwives as they step up their campaign for better pay. https://www.rcm.org.uk/media-releases/2020/november/deliver-a-decent-deal-say-midwives-as-they-step-up-their-campaign-for-better-pay/. Accessed 5 December 2020.

[CR42] Royal College of Midwives (RCM). 2020c. Fears for maternity as staffing shortages hit safety and morale says RCM. https://www.rcm.org.uk/media-releases/2020/november/fears-for-maternity-as-staffing-shortages-hit-safety-and-morale-says-rcm/. Accessed 1 December 2020.

[CR43] Royal College of Midwives (RCM). 2020d. RCM plea: help us deliver safe care for pregnant women. https://www.rcm.org.uk/media-releases/2020/march/rcm-plea-help-us-deliver-safe-care-for-pregnant-women. Accessed 5 April 2020

[CR44] Schopen, Fay. 2017. The healthcare gender bias: do men get better medical treatment?’ *The Guardian*, 20 November. https://www.theguardian.com/lifeandstyle/2017/nov/20/healthcare-gender-bias-women-pain. Accessed 29 November 2020.

[CR45] Shabot, Sara C. 2020. Why ‘normal’ feels so bad: violence and vaginal examinations during labour: a (feminist) phenomenology’. *Feminist Theory:* 1–21.

[CR46] Shepard Ashley (2010). The purple line as a measure of labour progress: A longitudinal study. BMC Pregnancy and Childbirth.

[CR47] Sherwood, Harriet. 2020. Midwife shortage doubles as NHS staff diverted to tend Covid-19 patients. *The Observer,* 29 March. https://www.theguardian.com/society/2020/mar/29/midwife-shortage-doubles-as-nhs-staff-diverted-to-tend-covid-19-patients. Accessed 5 April 2020.

[CR48] Stepney, Rob. 1994. When it’s the vagina use a personal touch. *The Guardian*, 19 April.

[CR49] Summers, Hannah. 2020a. Expectant mothers turn to freebirthing after home births cancelled. *The Guardian,* 5 April. https://www.theguardian.com/lifeandstyle/2020/apr/05/expectant-mothers-turn-to-freebirthing-after-home-births-cancelled. Accessed 10 April 2020.

[CR50] Summers, Hannah. 2020b. ‘Women feel they have no option but to give birth alone’: the rise of freebirthing. *The Guardian,* 5 December. https://www.theguardian.com/lifeandstyle/2020/dec/05/women-give-birth-alone-the-rise-of-freebirthing. Accessed 5 December 2020.

[CR51] The Birth Trauma Association. 2018. What is birth trauma? https://www.birthtraumaassociation.org.uk/for-parents/what-is-birth-trauma. Accessed 19 September 2020.

[CR52] Wolf Allison, Charles Sonya (2018). Childbirth is not an emergency: Informed consent in labor and delivery. International Journal of Feminist Approaches to Bioethics.

[CR53] World Health Organization (WHO). 2020. Reproductive Health: Pregnancy during Emergencies. https://www.who.int/reproductivehealth/publications/emergencies/Pregnancy-3-1200x1200.png?ua=1. Accessed 22 March 2020.

